# Supplementary material for: Depletion assisted hemin affinity (DAsHA) proteomics reveals an expanded landscape of heme-binding proteins in the human proteome
Source: Metallomics. 2023 Jan 20;15(3):mfad004. doi: 10.1093/mtomcs/mfad004 (PMC10022665; doi:10.1093/mtomcs/mfad004)
Supplement: mfad004_Supplemental_Files [file mfad004_supplemental_files.zip › Suppl_data_Kim_etal_2022_SUPPLEMENT_Accepted.docx]

**SUPPLEMENTAL INFORMATION**

**Depletion Assisted Hemin Affinity (DAsHA) Proteomics Reveals an Expanded Landscape of Heme Binding Proteins in the Human Proteome**

Hyojung Kim^1^, Courtney M. Moore^1^, Santi Mestre-Fos^1^, David A. Hanna^1^, Loren Dean Williams^1^, Amit R. Reddi^1^*, Matthew P. Torres^1,2^*

Contains Figures S1 – S6

**
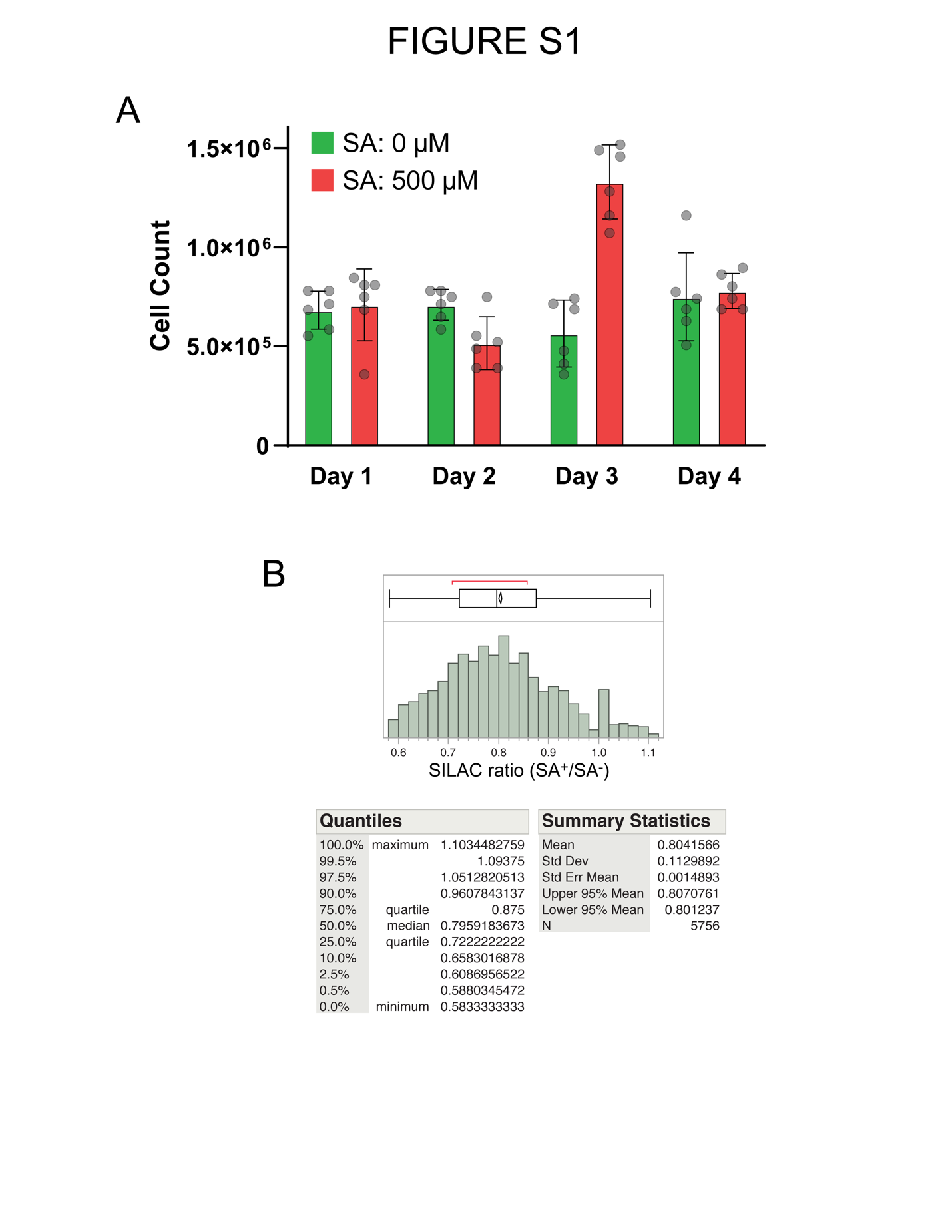
**

**Figure S1. Effects of succinylacetone treatment on cell survival and whole proteome abundance.** (A) Cell viability as measured by cell counts over 4 days of treatment with 500μM succinylacetone compared to controls. (B) SILAC ratios across 5756 unique proteins (representing 83% of all proteins observed) measured with (SA^+^) versus without (SA^-^) succinylacetone treatment. Extreme outliers (413) were removed for this analysis so as to reflect the bulk average effect of treatment on protein levels. A ratio of 1 equates to proteins whose abundance is identical between groups.


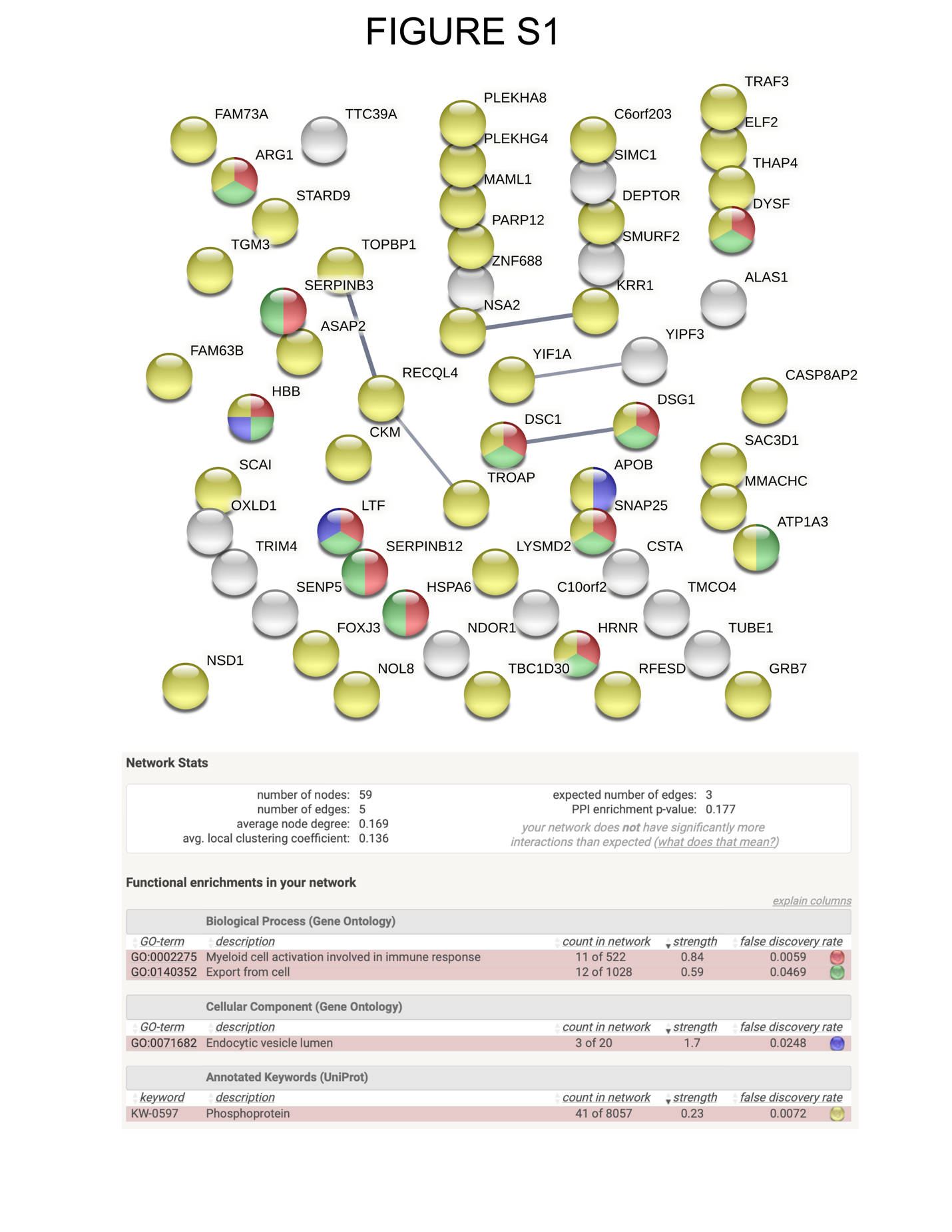


**Figures S2.** Proteins undergoing extreme abundance changes in response to heme depletion with succinylacetone. Only High confidence physical interactions are shown (grey lines).


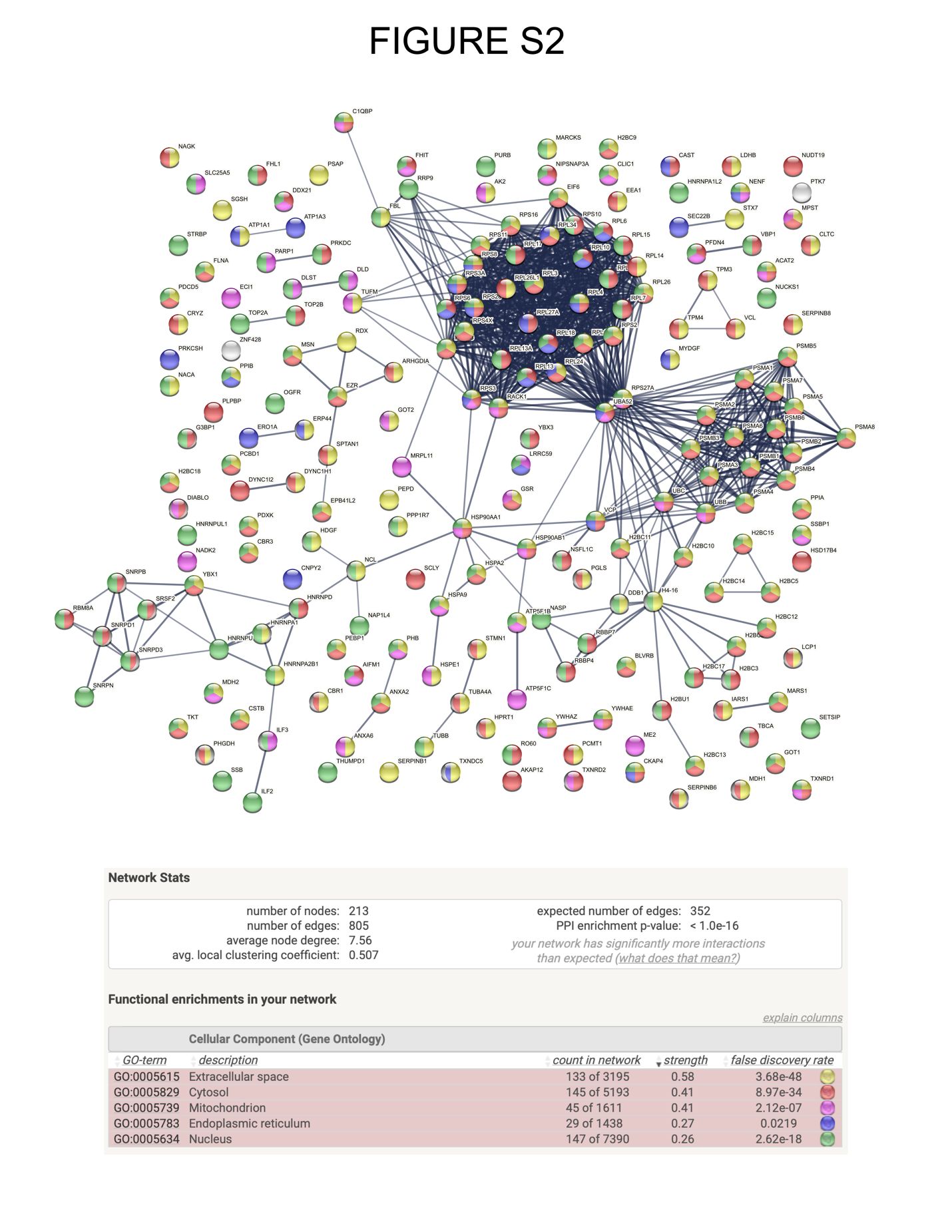


**Figure S3.** **The heme-binding proteins identified in this study localize to all major locations in the cell.** Full STRING network for proteins classified as moderate or high specificity hemin agarose binding in either SA- or SA+ conditions. Proteins are clustered by MCL method (default). Only high confidence physical interactions are shown (grey lines). Color coding key is show in table at bottom.


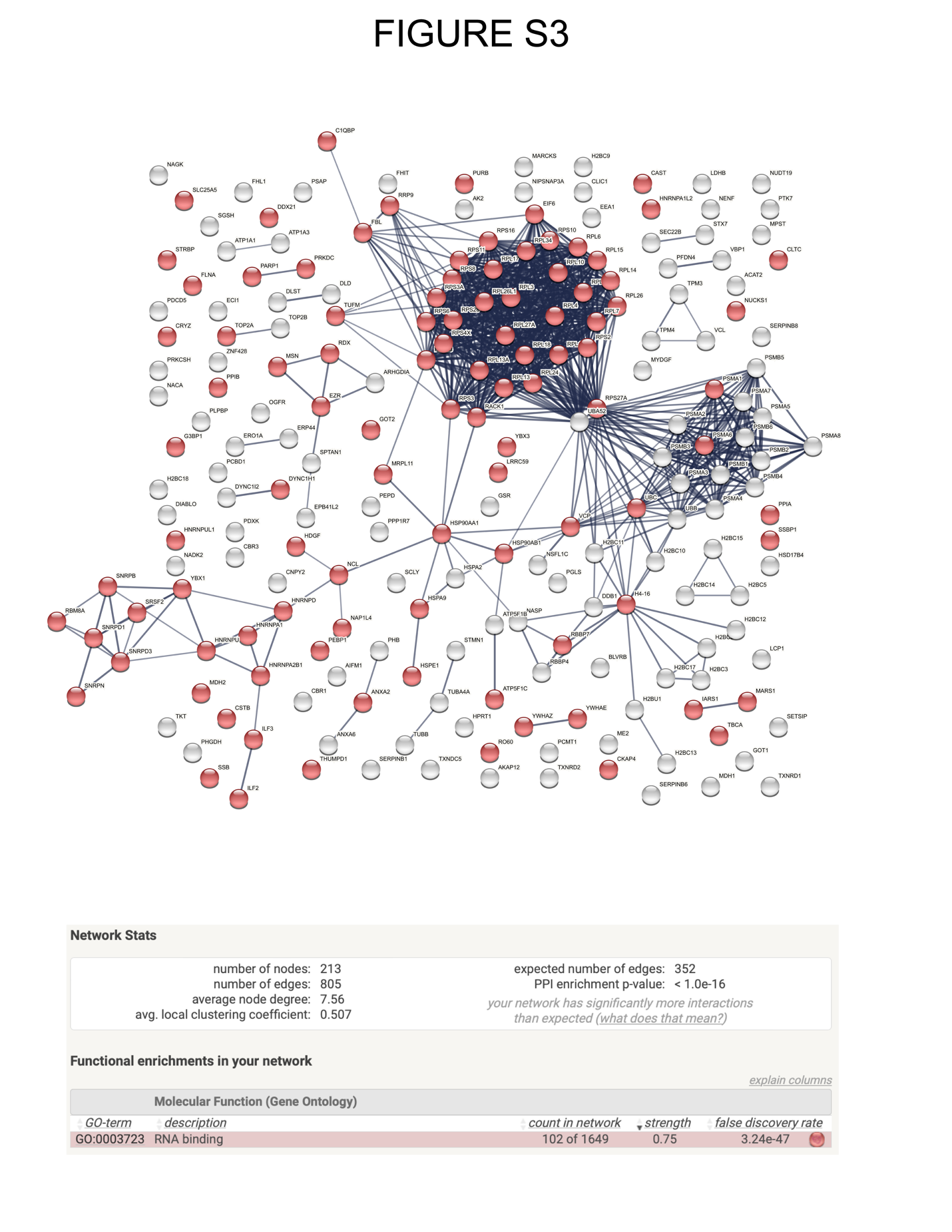


**Figure S4. Nearly 50% of all hemin agarose specific proteins are RNA binding proteins.** Full STRING network for proteins classified as moderate or high specificity hemin agarose binding in either SA- or SA+ conditions. Proteins are clustered by MCL method (default). Only high confidence physical interactions are shown (grey lines). Color coding key is show in table at bottom.


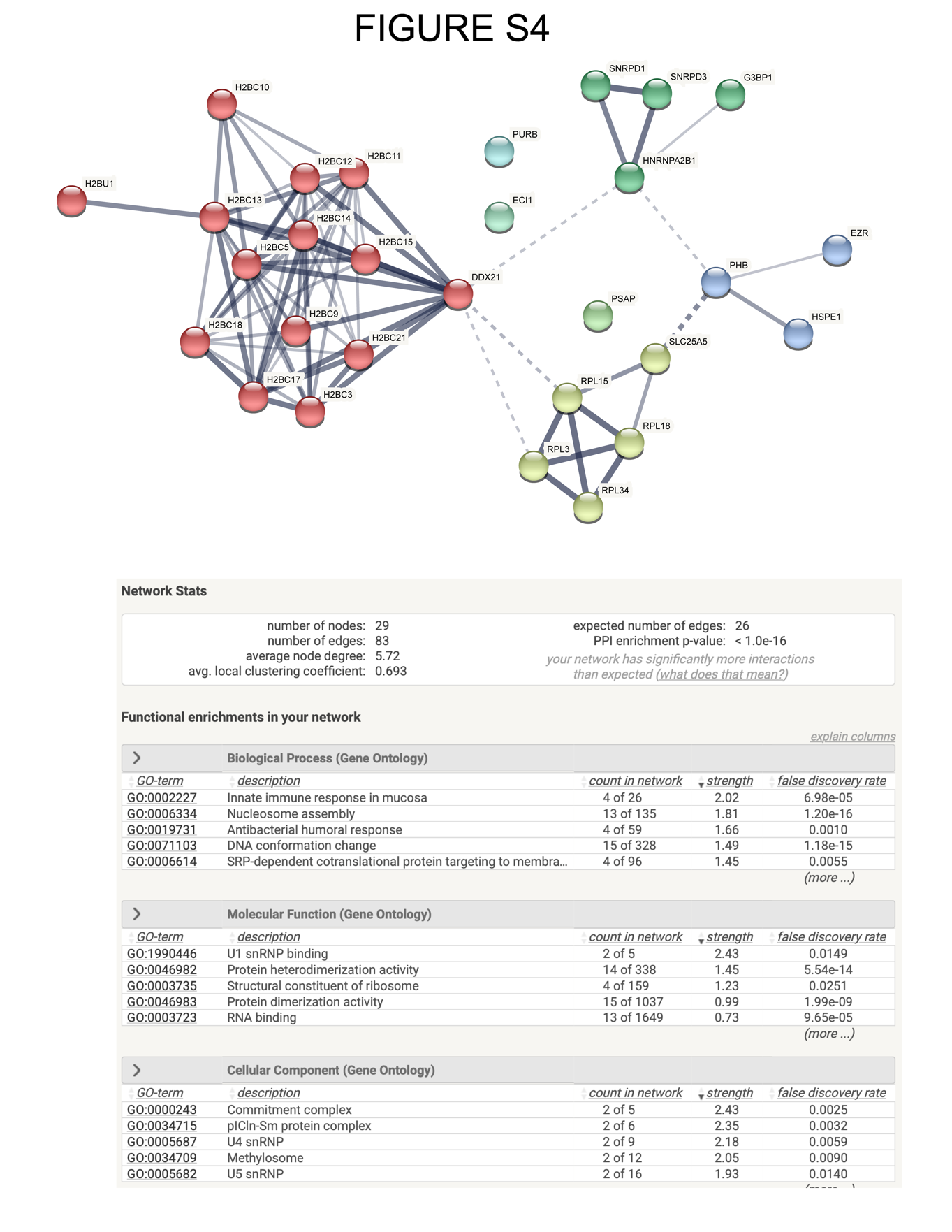


**Figure S5. Subset of hemin agarose-interacting proteins that also undergo extreme abundance change in response to heme depletion with succinylacetone.** Proteins shown are those with large change in abundance after SA treatment (Log_2_ SA+/SA- >5) that also undergo significant enrichment with hemin agarose compared to sepharose. Bottom table shows major GO terms for this network.


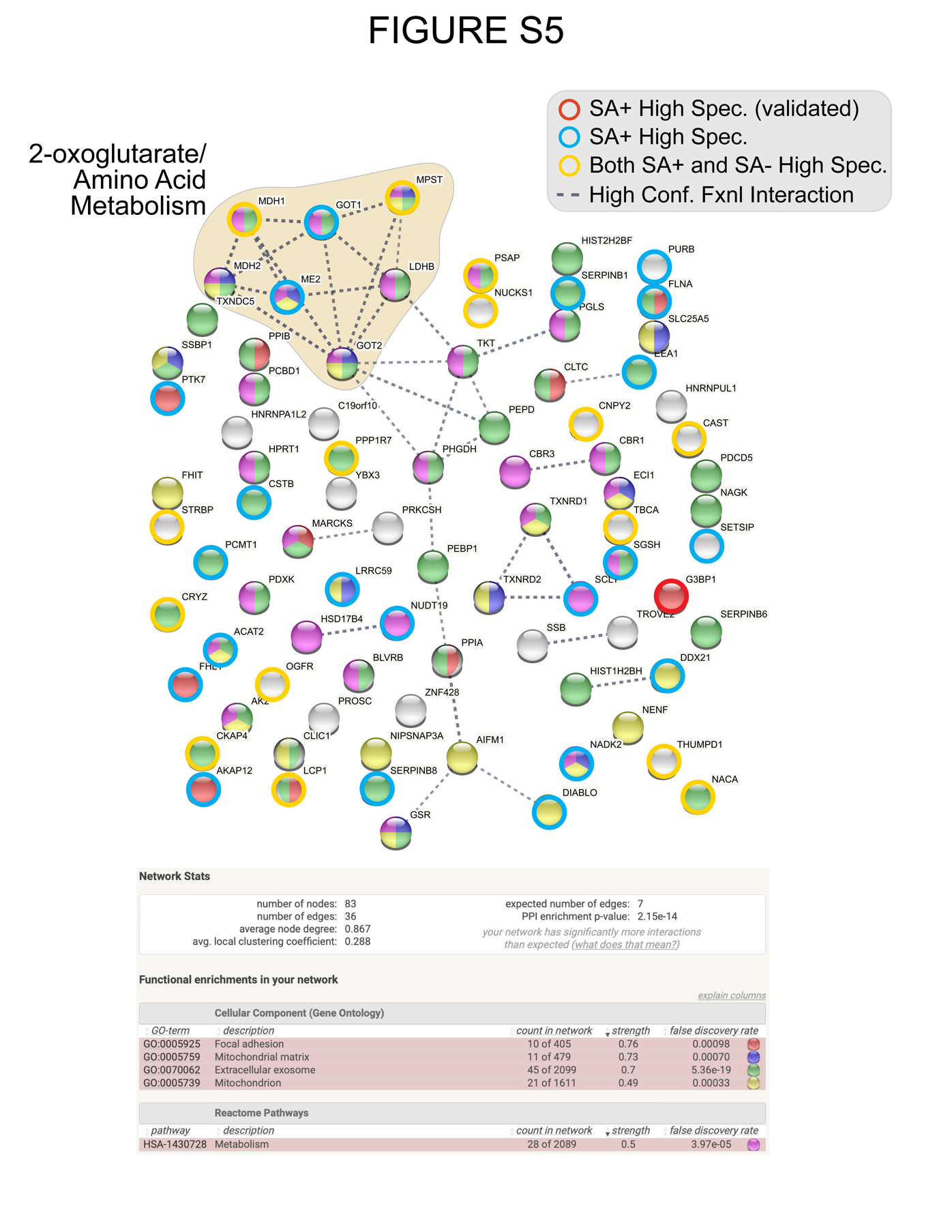


**Figure S6. Subset of hemin agarose-interacting proteins that do not exhibit high confidence physical interactions.** Proteins were clustered using the MCL inflation method in STRING. Spheres encircled with red, blue, or yellow rings were detected in the high specificity class (exclusively detected from hemin agarose but not sepharose enrichment) while spheres without rings were detected in the moderate specificity class (2 to 70-fold hemin agarose versus sepharose enrichment). Amino acid metabolism cluster is also shown in Figure 6.
